# Clinical and cognitive outcomes in first-episode psychosis: focus on the interplay between cannabis use and genetic variability in endocannabinoid receptors

**DOI:** 10.3389/fpsyg.2024.1414098

**Published:** 2024-08-12

**Authors:** Maitane Oscoz-Irurozqui, Maria Guardiola-Ripoll, Carmen Almodóvar-Payá, Amalia Guerrero-Pedraza, Noemí Hostalet, María Isabel Carrion, Salvador Sarró, JJ Gomar, Edith Pomarol-Clotet, Mar Fatjó-Vilas

**Affiliations:** ^1^FIDMAG Germanes Hospitalàries Research Foundation, Barcelona, Spain; ^2^Red de Salud Mental de Gipuzkoa, Osakidetza-Basque Health Service, Gipuzkoa, Spain; ^3^Centro de Investigación Biomédica en Red de Enfermedades Raras (CIBERER), Instituto de Salud Carlos III, Madrid, Spain; ^4^Centro de Investigación Biomédica en Red de Salud Mental (CIBERSAM), Instituto de Salud Carlos III, Madrid, Spain; ^5^Departament de Biologia Evolutiva, Ecologia i Ciències Ambientals, Facultat de Biologia, Universitat de Barcelona, Barcelona, Spain; ^6^Hospital Benito Menni CASM, C/Doctor Antoni Pujadas, Barcelona, Spain; ^7^Hospital Sant Rafael, Passeig de la Vall d’Hebron, Barcelona, Spain; ^8^The Litwin-Zucker Alzheimer's Research Center, The Feinstein Institutes for Medical Research, Manhasset, NY, United States

**Keywords:** first episode psychosis, cannabis, cannabinoid receptors genes (*CNR1* and *CNR2*), symptoms, cognition

## Abstract

**Introduction:**

Research data show the impact of the endocannabinoid system on psychosis through its neurotransmission homeostatic functions. However, the effect of the endocannabinoid system genetic variability on the relationship between cannabis use and psychosis has been unexplored, even less in first-episode patients. Here, through a case-only design, we investigated the effect of cannabis use and the genetic variability of endocannabinoid receptors on clinical and cognitive outcomes in first-episode psychosis (FEP) patients.

**Methods:**

The sample comprised 50 FEP patients of European ancestry (mean age (sd) = 26.14 (6.55) years, 76% males), classified as cannabis users (58%) or cannabis non-users. Two Single Nucleotide Polymorphisms (SNP) were genotyped at the cannabinoid receptor type 1 gene (*CNR1* rs1049353) and cannabinoid receptor type 2 gene (*CNR2* rs2501431). Clinical (PANSS, GAF) and neuropsychological (WAIS, WMS, BADS) assessments were conducted. By means of linear regression models, we tested the main effect of cannabis use and its interaction with the polymorphic variants on the clinical and cognitive outcomes.

**Results:**

First, as regards cannabis effects, our data showed a trend towards more severe positive symptoms (PANSS, *p* = 0.05) and better performance in manipulative abilities (matrix test-WAIS, *p* = 0.041) among cannabis users compared to non-users. Second, concerning the genotypic effects, the T allele carriers of the *CNR1* rs1049353 presented higher PANSS disorganization scores than CC homozygotes (*p* = 0.014). Third, we detected that the observed association between cannabis and manipulative abilities is modified by the *CNR2* polymorphism (*p* = 0.022): cannabis users carrying the G allele displayed better manipulative abilities than AA genotype carriers, while the cannabis non-users presented the opposite genotype-performance pattern. Such gene–environment interaction significantly improved the overall fit of the cannabis-only model (Δ-R^2^ = 8.4%, *p* = 0.019).

**Discussion:**

Despite the preliminary nature of the sample, our findings point towards the role of genetic variants at *CNR1* and *CNR2* genes in the severity of the disorganized symptoms of first-episode psychosis and modulating cognitive performance conditional to cannabis use. This highlights the need for further characterization of the combined role of endocannabinoid system genetic variability and cannabis use in the understanding of the pathophysiology of psychosis.

## 1 Introduction

Schizophrenia (SZ) is a complex psychiatric disorder with a lifetime prevalence of 5.5/1000 (McGrath et al., 2008). Positive symptoms include hallucinations, delusions and disorganized thought (manifested in speech and behavior); while negative symptoms include blunted affect, anhedonia, alogia, avolition and social withdrawal. SZ is also characterized by cognitive deficits affecting memory, attention, and executive functions. These manifestations are associated with functional and social impairment, placing SZ as a major cause of disability worldwide (Murray et al., 1996).

The current knowledge indicates that the origin of SZ must be understood by the combination of multiple genetic and environmental factors (Zwicker et al., 2018). Among different environmental factors, cannabis use has been consistently associated with the risk for psychosis (Di Forti et al., 2014; Marconi et al., 2016). Its use is associated with a 2-fold risk for schizophrenia-spectrum disorders, which increases up to almost a 4-fold risk in cannabis heavy users (Marconi et al., 2016).

In a systematic review and meta-analysis, cannabis use in patients with SZ and other psychotic disorders has been linked to more severe positive symptoms (Schoeler et al., 2016). Particularly in early psychosis, some studies have also reported that the severity of psychotic symptoms is higher in cannabis users both in positive (Grech et al., 2005; Baeza et al., 2009; Seddon et al., 2016) and negative symptoms (Seddon et al., 2016), while others did not find any effect of cannabis on psychotic symptomatology (Barrowclough et al., 2015).

Focusing on cannabis effects on cognition, review works that summarize evidence in healthy subjects conclude that cognitive deficits associated with cannabinoids (both acute and chronic exposure) are rather global, with the domains of verbal memory, attention and executive functioning being affected most consistently (Broyd et al., 2016; Cohen and Weinstein, 2018). Regarding studies based on patients with a diagnosis within the psychosis spectrum, some studies describe a worse cognitive performance associated with cannabis use (Núñez et al., 2016; Bogaty et al., 2018); others show a better performance (Løberg and Hugdahl, 2009; Yücel et al., 2012; Schoeler et al., 2016), and there are also studies showing no effect (Bugra et al., 2013). Such diverse results must be accounted by several social, clinical and cannabis composition factors but may also be related to the biologically influenced sensitiveness to the cannabis effects (Henquet et al., 2008).

In search for the biological mechanisms responsible for these heterogeneous effects of cannabis use on psychosis, several lines of evidence have implicated the endocannabinoid system (ECS) [for a review, see (Garani et al., 2021)]. The ECS is mainly composed of endogenous ligands and their receptors [the predominantly central cannabinoid receptor type 1 (CB1R) and mostly peripheral type 2 receptor (CB2R)]. The endocannabinoid system functions by engaging with various neurotransmission systems and regulating numerous cognitive and emotional reactions within the central nervous system, thereby contributing to maintaining brain homeostasis (Wolf et al., 2008; Ibarra-Lecue et al., 2018). Exogenous cannabinoids bind to cannabinoid receptors, and, in that way, cannabis use can disturb physiological control of the endogenous cannabinoid system over the release of other neurotransmitters. This may ultimately lead to the development of psychosis or SZ depending on frequency, dose, and brain maturation status at exposure (Bossong and Niesink, 2010), as well as on the individual genetic background (Bioque et al., 2019).

Specifically, the cannabis effects are mediated by its main psychoactive component, ∆^9^-THC, which is a partial agonist of ECS receptors. The genes encoding CB1R and CB2R (*CNR1* and *CNR2,* respectively), have been proposed as candidate genes associated with psychosis and SZ. However, there is conflicting evidence regarding whether different polymorphisms in these genes are associated with specific clinical and cognitive phenotypes of psychosis.

On the one hand, different studies have shown associations between *CNR1* genetic variants and the psychosis outcome, including positive (Çöpoğlu et al., 2015; Suárez-Pinilla et al., 2015), negative (Ujike et al., 2002; Chavarría-Siles et al., 2008; Çöpoğlu et al., 2015; Suárez-Pinilla et al., 2015) and disorganized symptoms (Chavarría-Siles et al., 2008; Çöpoğlu et al., 2015). Van Winkel et al. (2011), focusing on unaffected siblings of patients with SZ as obligate carriers of genetic vulnerability, found significant associations between *CNR1* SNPs and schizotypal symptoms and signs, as well as a *CNR1* × cannabis recent use interaction effect.

Literature about *CNR2* genetic variability in psychosis is scarce, and it is mainly based on case–control association analyses about the risk of developing SZ (Ishiguro et al., 2010; Tong et al., 2013; EO et al., 2014). Also, a study based on a sample of healthy individuals reported an effect of *CNR2* on distressing psychotic experiences, such as visual hallucinations, auditory hallucinations, delusions of reference or delusions of persecution (Legge et al., 2019).

Genetic variants of *CNR1* have also been associated with cognitive performance in several dimensions, but most studies have been carried out in healthy individuals (Ruiz-Contreras et al., 2011, 2013, 2014, 2017; Colizzi et al., 2015; Taurisano et al., 2016). Remarkably, an interaction effect between cannabis use and the CB1-related co-expression gene network on dorsolateral prefrontal activity during working memory performance has been described in healthy subjects (Taurisano et al., 2021). Also, the interplay between cannabis use and *CNR1* genetic variants has been reported on brain volumes in healthy subjects (Hill et al., 2016). On the other hand, only few studies have evaluated such effects in psychotic patients. Cross-sectional studies describe associations between certain *CNR1* polymorphisms and cognitive performance across different domains in SZ patients (Ho et al., 2011; Ferretjans et al., 2022). In subjects with a first-episode of psychosis (FEP), another *CNR1* polymorphism was associated with differential improvements in verbal memory and attention after 18 months of treatment (Rojnic Kuzman et al., 2019). Also, it has been shown the role of cannabis exposure as a moderating factor in the link between *CNR1* genotypes and neurocognitive measures in patients with SZ (Ho et al., 2011).

As far as we know, no studies have comprehensively investigated the effects of *CNR2* on clinical and neurocognitive phenotypic features in FEP patients, nor its interaction with cannabis use. A piece of evidence comes from a study that found that one polymorphism at *CNR2* was nominally associated with the performance of a working memory test in patients with SZ (Ferretjans et al., 2022). Also, different authors have indicated the role of *CNR2* in memory function in animal model-based studies using knock-out specimens or assessing CB2R expression (Wu et al., 2013; Li and Kim, 2017; Schmöle et al., 2018).

The aforementioned points underscore the need for extensive research into the impact of cannabis use on FEP outcomes, as well as the involvement of cannabinoid receptors in modulating these effects. Then, by means of a case-only study design, we aimed to investigate whether cannabis use and genetic variants at ECS receptor genes (and their interplay), influence the symptoms, the functionality outcome, and the cognitive performance in patients with a FEP.

## 2 Materials and methods

### 2.1 Subjects

The study involved 50 patients experiencing their first non-affective psychosis episode, recruited at the Benito Menni CASM Hospital and Sant Rafael Hospital, employing consecutive sampling methods. Each patient was suffering from the first episode of psychosis, with symptom duration not exceeding 18 months. The participant pool consisted of adults (aged 18 years or older) of European descent. This sample partially overlaps with the cohort described in a prior research article by our group (Oscoz-Irurozqui et al., 2023), which focused on the analysis of the working memory neural correlates (assessed by functional magnetic resonance), taking into account cannabis use and cannabinoid receptor genes.

Exclusion criteria comprised: (i) age exceeding 65 years, (ii) premorbid Intelligence Quotient (IQ) below 75, (iii) a documented history of brain trauma resulting in loss of consciousness or any neurological condition, and (iv) the existence of a DSM-IV affective psychotic diagnosis, including mania, hypomania, and major depression with psychotic symptoms.

After 6 months, patients underwent a diagnostic assessment employing the Spanish version of the Structured Clinical Interview for DSM-IV (SCID). The diagnoses were: schizophrenia (*n* = 22), schizoaffective disorder (*n* = 3), delusional disorder (*n* = 1), and unspecified psychosis (*n* = 14).

### 2.2 Clinical and cognitive assessments

Symptoms were scored using the patients’ clinical evaluation, which included the Positive and Negative Symptoms Scale (PANSS) (Kay et al., 1987). Based on the PANSS, Positive, Negative and Disorganized Syndrome scores were calculated (Wallwork et al., 2012). We also administered the Global Assessment of Functioning (GAF) scale (Jones et al., 1995), as a single measure of the severity of illness and the overall psychosocial impairment.

The pre-morbid IQ was determined with the Word Accentuation Test (Test de Acentuación de Palabras, TAP) (Gomar et al., 2011), a word reading test requiring pronunciation of Spanish words whose accents have been removed. Patients also performed four subtests of the Wechsler Adult Intelligence Scale III (WAIS-III: vocabulary, similarities, block design, and matrix reasoning) to assess their current verbal and manipulative IQ.

Two well-standardized tests of executive function and memory were also administered. The Behavioural Assessment of the Dysexecutive Syndrome (BADS) (Wilson et al., 1996) gives an overall ‘profile’ score based on performance on six different subtests. The other consisted of four subtests of the Wechsler Memory Test (WMS-III (Del et al., 1997) logical memory, faces, digit span, letter-number sequencing); scaled scores on these subtests were summed to give an overall score. Diagnostic evaluation and clinical and neuropsychological assessments were carried out by an experienced psychiatrist and psychologist, respectively.

### 2.3 Cannabis use

The use of recreational drugs was obtained through patients’ self-reports during interviews and on medical records. Patients with alcohol/substance (except cannabis) abuse or dependence within the 6 months before participation were excluded.

Assessment of cannabis use spanned each subject’s lifetime. Those classified as cannabis non-users either never used cannabis or had only experimented with it once. On the other hand, cannabis users were identified as individuals with consistent cannabis consumption, with a significant majority (85%) meeting the Diagnostic and Statistical Manual of Mental Disorders (DSM-IV-TR) criteria for cannabis abuse or dependence. At the time of clinical and cognitive evaluations, participants had abstained from cannabis for at least 1 week.

### 2.4 Genetic data

Genomic DNA extraction was performed for all individuals, sourced either from buccal mucosa using cotton swabs and the ATP Genomic DNA Mini Kit Tissue (Teknokroma Analítica, S.A., Sant Cugat del Vallès, Barcelona, Spain) or from peripheral blood cells using the Realpure SSS Kit for DNA Extraction (Durviz, S.L.U, Valencia, Spain).

Two single nucleotide polymorphisms (SNPs) were genotyped: rs1049353 located at *CNR1* (Chr: 6q14-q15) and rs2501431 at *CNR2* (Chr: 1p34-p35) genes. These specific SNPs were chosen based on: (i) previous research indicating their potential association with psychosis and/or cannabis use (Costa et al., 2013; Suárez-Pinilla et al., 2015; Hill et al., 2016; Gerra et al., 2018), (ii) Minor Allele Frequency (MAF) in the European population higher than 10%. The genotyping process employed a fluorescence-based allelic discrimination procedure, specifically the Applied Biosystems Taqman 5′-exonuclease assays, conducted under standard conditions. To verify accuracy, 15 samples underwent re-genotyping, with all repeated genotypes matching the initial results. Genotype frequencies demonstrated conformity to Hardy–Weinberg equilibrium. For analysis purposes, genotypes were dichotomized as follows: *CNR1* rs1049353 (CC vs. T-carrier) and *CNR2* rs2501431 (AA vs. G-carrier).

### 2.5 Statistical analyses

The analyses were conducted using SPSS 23.0 software (IBM SPSS Statistics for Windows, version 23.0, released in 2015, IBM Corporation, Armonk, New York).

Demographics and genotypic data of cannabis users and cannabis non-users were compared through t-student and chi-square tests.

Linear regressions were used to test the effects of cannabis use, genotype, and cannabis × genotype interaction on clinical and cognitive data. All the analyses were covaried by age and sex to control for the potential confounding effects of these variables. We used for those regressions a 3-step model including previous factors as independent variables, successively added to it (model 1: cannabis; model 2: cannabis + genotype; model 3: cannabis + genotype + interaction cannabis × genotype). Finally, when the interaction effect was found, we performed the likelihood-ratio test to assess the goodness of fit of the two statistical models, to compare the additive and interaction effects on the dependent variable. We show standardized values of the statistical parameters.

For the between-groups *post-hoc* statistical power calculation, we used G*Power 3.1.9 (Faul et al., 2009). As regards the clinical severity, functionality and cognitive performance comparisons, both between cannabis users and non-users and between genotypes, our sample was powered (1-β = 0.80, α = 0.05) to detect large effect sizes (d > 0.75). As an example, it corresponds to 4 points on PANSS positive syndrome scores or 3 points on the WAIS matrix subtest between cannabis users and non-users, or 2 points on PANSS disorganized syndrome scores between T allele carriers and non-carriers of the *CNR1* polymorphism. For the interaction models, the *post-hoc* statistical power was assessed using the ‘pwr’ R package. It showed that our sample was powered to detect medium effect sizes (*d* > 0.17).

## 3 Results

### 3.1 Sample description

Twenty-nine (58%) participants were classified as cannabis users. There were no significant differences in demographic characteristics, premorbid IQ and medication dosage between users and non-users (Table 1).

**Table 1:** Sociodemographic data and genotype distribution between Cannabis Non-Users (CNU) and Cannabis Users (CU) of the first-episode psychosis patients included in the study.

	CNU	CU	CNU *vs* CU comparison
			*p*-value
N	21	29	–
Age (years)^a^	26.36 (7.55)	25.15 (4.84)	0.523
Sex (male/female)	15/6	23/6	0.089
Premorbid IQ (TAP)^b^	97.23 (11.29)	98.64 (7.17)	0.654
CPZ equivalents (mg/day)^c^	285.91 (144.84)	321.98 (194.25)	0.476
*CNR1* (rs1049353): CC genotype	15	17	0.352
*CNR1* (rs1049353): TT/TC genotypes	6	12	
*CNR2* (rs2501431): AA genotype	6	12	0.352
*CNR2* (rs2501431): GG/GA genotypes	15	17	

Statistical comparisons were conducted using chi-square and *t*-test when appropriate. N = sample size. IQ = Intelligence Quotient. TAP=Word Accentuation Test. CPZ = Chlorpromazine. All the quantitative variables include the mean value (standard deviation).

a Age range 18–39 years.

b Data of TAP were available for 46 patients.

c All patients except 2 were on antipsychotic treatment when the tests were performed.

The genotypic distribution of the rs1049353 and rs2501431 genotypes is shown in Table 1. Minor allele frequencies were T = 0.21 and G = 0.4, respectively, in line with those described for the 1,000 Genomes EUR super population. After genotype determination, the two SNPs displayed Hardy–Weinberg equilibrium in both groups (*p* > 0.05). No genotype distribution differences were observed between groups.

### 3.2 Cannabis use and genotypic effects on symptoms severity and functionality

Cannabis users showed a trend towards higher PANSS positive syndrome scores than non-users (ß = 0.289; SE = 1.482; *p* = 0.050; R^2^ = 0.028; Table 2). As regards the other clinical parameters, including GAF scale, no significant differences between cannabis use groups emerged (Table 2).

**Table 2:** Clinical outcomes comparison between Cannabis Non-Users (CNU) and Cannabis Users (CU), through linear regression models (covaried by age and sex).

	CNU	CU	CNU *vs* CU comparison
			***p*-value**
Positive syndrome – PANSS	13.86 (4.93)	16.75 (5.05)	**0.050**
Negative syndrome – PANSS	14.24 (8.36)	15.07 (7.41)	0.956
Disorganized syndrome – PANSS	7.24 (2.64)	8.21 (2.50)	0.210
GAF	52.53 (10.95)	47.25 (10.91)	0.088

PANSS, Positive and Negative Syndrome Scale; GAF, Global Assessment of Functioning. The mean values and standard deviation are reported.

The polymorphic variant at the *CNR1* was associated with the PANSS disorganized syndrome scores (ß = 0.337; SE = 0.705; *p* = 0.014; R^2^ = 0.221). Individuals with TT/TC genotypes showed higher mean (sd) scores [9.29(2.08)] than those with the CC genotype [7.00(2.49)] (see Supplementary Tables S1, S2 for full model data).

There was no effect of *CNR2* gene neither on psychotic symptoms or functionality.

### 3.3 Cannabis use and genotypic effects on cognitive performance

As regards cannabis use, it significantly modulated the performance score on the matrix test of WAIS (Table 3). In particular, cannabis users showed better scores in comparison with non-users (ß_standardised_ = 0.323; SE = 1.144, *p* = 0.041, R^2^ = 0.040). No other significant differences between groups emerged on cognitive performance according to the used tests (Table 3).

**Table 3:** Cognitive performance comparison between Cannabis Non-Users (CNU) and Cannabis Users (CU), through linear regression models (covaried by age and sex).

	CNU	CU	CNU *vs* CU comparison
			*p*-value
Vocabulary test – WAIS	9.77 (2.82)	9.19 (1.96)	0.478
Similarities test – WAIS	9.29 (2.85)	9.27 (2.28)	0.953
Matrix test – WAIS	7.06 (4.04)	9.41 (3.31)	**0.041**
Block design test – WAIS	7.94 (3.11)	8.82 (2.80)	0.373
Manipulative IQ	82.65 (17.46)	92.74 (16.35)	0.063
Verbal IQ	95.24 (16.94)	93.58 (11.76)	0.788
BADS	14.40 (4.27)	17.08 (3.84)	0.071
WMS	26.19 (9.03)	28.63 (6.34)	0.259

WAIS, Wechsler Adult Intelligence Scale; BADS, Behavioural Assessment of the Dysexecutive Syndrome; WMS, Wechsler Memory Scale. Mean scores and standard deviation of scalar scores are reported.

**Figure 1:**
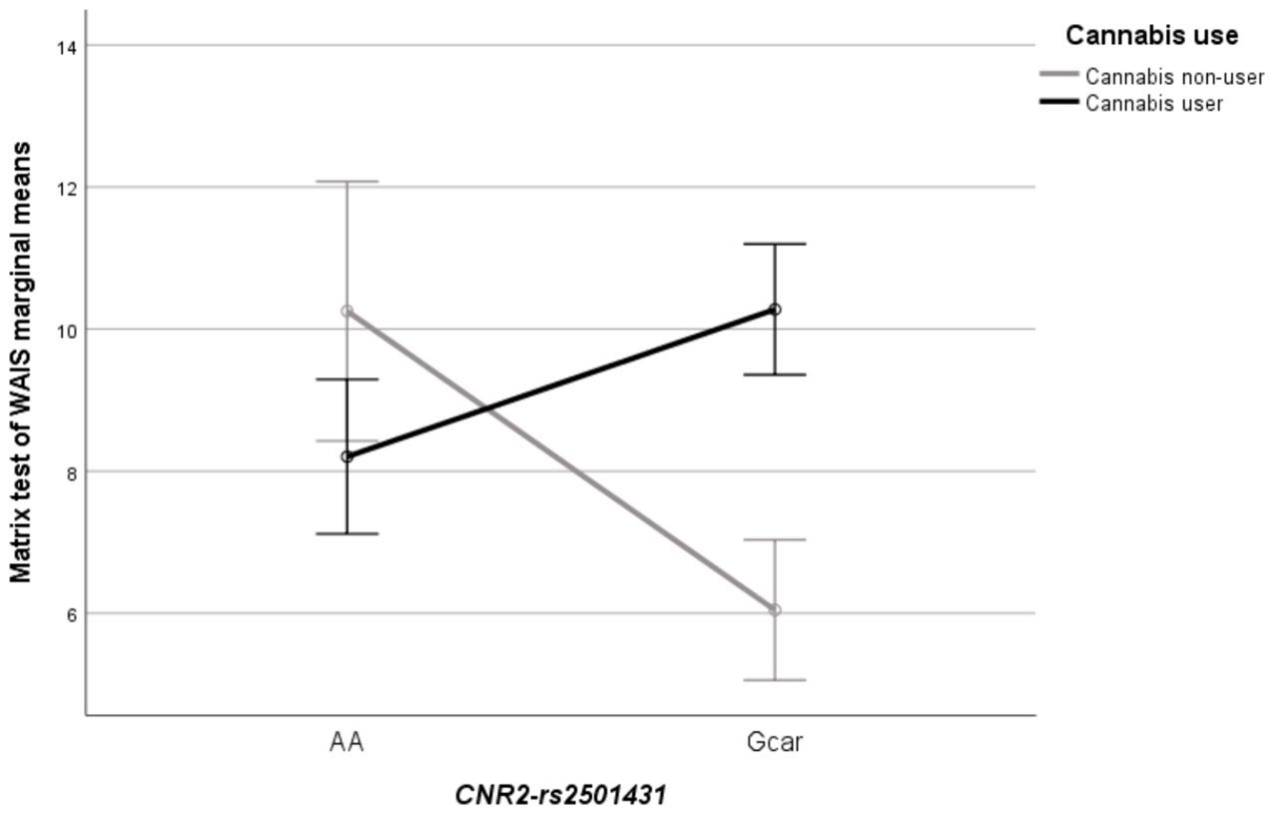
Line plot showing the significant interaction between *CNR2* (rs2501431) × cannabis use (cannabis non-users; cannabis users) in the matrix test of WAIS. Each bar represents the marginal mean of the test score (±1SE), separately by genotypes and cannabis use groups.

There was no main effect of cannabinoid receptor genes on cognitive scores, while the interaction models showed an interplay between *CNR2,* and cannabis use for the matrix test (ß = 0.728; SE = 2.374; R^2^ = 0.124; *p* = 0.022). Adding the interaction term in a stepwise manner improved the model’s overall fit (Δ-R^2^ = 8.4%, *p* = 0.019). Such interaction indicates that the genotype effect was conditional to cannabis use: within cannabis non-users, individuals with the AA genotype showed better manipulative abilities [10.00 (5.89)] than G-allele carriers [6.15 (3.05)]; while, within cannabis users, G-allele carriers performed the test better [10.19 (3.21)] than the AA homozygotes [8.27 (3.26)] (Figure 1).

## 4 Discussion

Our results show the independent but also the joint effect of cannabis use and cannabinoid receptors’ genetic variants on the outcome of first psychosis. In this regard, this study supports the growing interest in understanding the role of both cannabis and genetic modulation effects on first-episode psychosis, to improve the knowledge of the pathophysiological mechanisms that can be targeted with preventive aims.

First, as regards the effect of cannabis use on symptoms, functionality and cognitive performance, our findings add to previous knowledge in a quite convergent manner. On the one hand, we report a tendency of more severe positive symptoms among cannabis users as compared to non-users. This effect was also shown in a large cohort of FEP patients, in which cannabis use was associated with higher PANSS positive scores, both at baseline and in the 12-month follow-up (Seddon et al., 2016). Also, these data are aligned with a systematic review and meta-analytic study, which concluded that continued cannabis use after the onset of psychosis predicts more severe positive symptoms than individuals who discontinue cannabis use or those who are non-users (Schoeler et al., 2016).

On the other hand, our results show a better manipulative performance in FEP patients cannabis users than non-users, joining the controversial already existing results. As previously mentioned, different studies about the cannabis effects on cognitive performance in patients with a recent onset of psychosis report heterogeneous results (Mata et al., 2008; Bugra et al., 2013; Bogaty et al., 2018). But paradoxically, it has been suggested in several studies that patients with SZ or FEP who are cannabis users display better cognitive functioning as compared with non-users (Løberg and Hugdahl, 2009; Schnell et al., 2009; Leeson et al., 2012; Cunha et al., 2013; Ferraro et al., 2013). This has been interpreted as that cannabis users could represent a subgroup of patients neurocognitively less damaged and/or with lower intrinsic vulnerability, in which the early start of cannabis consumption would have triggered the onset of psychosis (Løberg and Hugdahl, 2009; Yücel et al., 2012). In addition, some studies show that patients with schizophrenia who consume cannabis have better social functioning when compared with non-users (Carey et al., 2003; Salyers and Mueser, 2003). However, we acknowledge that other complementary explicative factors could be participating, such as the type of cannabis or the frequency of use, as well as the genetic background of each person.

Second, regarding the analyzed genetic variants, our data indicate a role of the polymorphism rs1049353 at the *CNR1* gene on the disorganized syndrome. These results would be in line with other findings highlighting the effect of *CNR1* variability on clinical profiles, which have described an association of the (AAT)n *CNR1* microsatellite with disorganized and negative symptoms on hebephrenic SZ in different populations (Ujike et al., 2002; Chavarría-Siles et al., 2008). In line with these results, the polymorphism *CNR1* rs6454674 has been associated with the psychotic symptoms in the PANSS subscales (PANSS total, PANSS positive, PANSS negative and PANSS general psychopathology) and Clinical Global Impression Severity Scale (CGI-S) (Çöpoğlu et al., 2015). The effect of *CNR1* has also been explored from neurobiological approaches. For instance, some neuroimaging-based studies have reported that different allelic variants of this gene impact different neuroanatomical structures, such as the caudate and thalamus or white matter volumes, in patients with SZ and FEP patients (Ho et al., 2011; Suárez-Pinilla et al., 2015). On the other hand, contrary to two previous studies in healthy subjects and patients with SZ (Ho et al., 2011; Colizzi et al., 2015), we did not find an interaction effect of the *CNR1* gene and cannabis use on cognition.

Concerning the *CNR2* gene, we did not observe any main effect on clinical outcomes or cognition. A review of the evidence coming from various animal models based on the *CNR2* gene and SZ-related symptoms (where schizophrenia-like symptoms were induced via CB2R modulation) concluded that this receptor plays a significant role in the regulation of anxiety- and depressive-related behaviors, cognition and locomotion, all of which are intimately related with the symptoms of SZ (Banaszkiewicz et al., 2020). Regarding cognition, in our line, Ferretjans et al. (2022) did not find an association between *CNR2* polymorphisms and cognitive performance in SZ patients. However, in animal models of SZ (through glutamatergic dysfunction induced by the NMDAR antagonist MK 801), the administration of a competitive CB2R antagonist (AM 630) exacerbated memory impairment and CB2R activation by a CB2R selective agonist (JWH 015) reversed cognitive impairment after MK 801 administration (Ishiguro et al., 2010; Khella et al., 2014), suggesting the role of CB2R in the cognitive impairment found among patients with SZ.

On the other hand, despite not finding a *CNR2* gene isolated effect on cognition, we described an interaction between the *CNR2* polymorphisms and cannabis use on the WAIS matrix test. In fact, our full model indicates a cannabis use effect (first step of the statistical model) but not a gene effect *per se* (second step of the additive statistical model). In this regard, it is noteworthy that within G-carriers, differences existed in the matrix test performance between cannabis users and non-users, while within AA individuals, the test scores were not so diverse. These results could reflect a differential cannabis impact depending on genetics, or, in other words, that genotype modulates cannabis sensibility, which affects cognitive outcome. Similarly, there are several pieces of literature regarding psychosis that show this modulating effect of genetics on cannabis effect, but also on other environmental factors (Henquet et al., 2008; Pelayo-Terán et al., 2012; Tomassi and Tosato, 2017; Zwicker et al., 2018; Wahbeh and Avramopoulos, 2021). For cannabis use, for example, Colizzi et al. (2015) in a case (FEP patients)-control study point to the impact of lifetime cannabis use on the susceptibility to developing a psychotic disorder, as well as the propensity for experiencing psychosis-like symptoms and cognitive alterations, which varies depending on the *DRD2* rs1076560 genotype (a gene that encodes the D2 subtype of the dopamine receptor). In another study (Estrada et al., 2011) on individuals with schizophrenia-spectrum disorders and other non-psychotic disorders, the *COMT* Val158Met genotype (a gene involved in catabolizing catecholamines such as dopamine) appears to modulate the relationship between cannabis use and the age at onset of psychotic disorders.

In addition, to further understand the impact of our results, it is interesting to add some functional data on the role of *CNR2* genetic variants. In this sense, Ishiguro et al. (2010) described the association of two SNPs near the *CNR2* locus with SZ in Japanese populations, and, remarkably, by means of gene expression assays, they showed the link between the genetic variants of risk with changes in the functional response of the CB2 receptor to its ligands. Accordingly, while aware of the scarcity of data, we could hypothesize that the interaction effect we detect could be similarly explained by changes in the gene expression derived from the polymorphic variant, which, in turn, would impact the sensitivity to cannabis use effects.

Summarizing, our study supports the cannabis and endocannabinoid system genetics role in the pathophysiology of SZ. However, it is important to interpret our findings in light of certain limitations. Firstly, cannabis use was identified through self-reports and medical records, and it was treated as a dichotomized variable. While previous studies have widely used this classification (Løberg and Hugdahl, 2009; Yücel et al., 2012; Suárez-Pinilla et al., 2015), it is worth noting that gathering more extensive data, including information on variables such as the percentage of THC, age at onset, frequency of use, method of consumption, and others, would be beneficial for future investigations. Also, we are aware that our comprehension of the causal interactions among pertinent factors for psychosis would be enhanced by a longitudinal design (Bioque et al., 2019). However, it is remarkable that the FEP-based sample allows minimizing the impact of illness duration-related factors as well as the cannabis effect after psychosis outcome. Furthermore, we acknowledge that our sample, due to its pilot nature, is underpowered for small-size effects; highlighting the need for new studies to validate both the positive and negative reported results. As regards the sample composition, we also recognize the imbalanced sex-ratio towards more males than females, which is a common issue when samples are recruited based on consecutive hospitalizations. Recent research has demonstrated the sex-dependent effects of cannabis and the ECBS (Spindle et al., 2021; Coleman et al., 2022); nevertheless, due to the composition of our sample, we were unable to conduct sex-specific analyses. Therefore, our analyses should be compared with previous studies based on mixed samples with a predominance of males (Løberg et al., 2012; Suárez-Pinilla et al., 2015). Finally, the absence of a control group limits interpretations regarding the influence of cannabis use and genetic factors on the initial stages of psychotic disorders and also excludes analyses concerning health-disease status and vulnerability.

In conclusion, our study indicates that both endocannabinoid system genetic variants and cannabis use may contribute to a differential outcome in patients with a first episode of psychosis. Despite larger samples would be necessary to better understand the origin of psychosis, current approaches are important to improve our knowledge of causal interactions between relevant factors, which could lead to personalized prevention efforts and therapies for clinicians.

## Data availability statement

The dataset generated for this study is available on request to the corresponding authors.

## Ethics statement

The study was conducted according to the guidelines of the Declaration of Helsinki, and ethical approval was obtained from the Clinical Research Ethics Committee of Hermanas Hospitalarias. All participants provided written consent subsequent to comprehensive information about the study’s procedures and implications.

## Author contributions

MO-I: Conceptualization, Data curation, Formal analysis, Investigation, Methodology, Writing – original draft, Writing – review & editing, Visualization. MG-R: Conceptualization, Data curation, Formal analysis, Investigation, Methodology, Writing – review & editing. CA-P: Conceptualization, Data curation, Formal analysis, Investigation, Methodology, Writing – review & editing. AG-P: Investigation, Methodology, Resources, Writing – review & editing. NH: Data curation, Methodology, Writing – review & editing. MC: Investigation, Writing – review & editing. SS: Investigation, Methodology, Writing – review & editing. JG: Investigation, Writing – review & editing. EP-C: Funding acquisition, Investigation, Methodology, Resources, Writing – review & editing. MF-V: Conceptualization, Data curation, Formal analysis, Funding acquisition, Investigation, Methodology, Project administration, Resources, Supervision, Writing – original draft, Writing – review & editing.
