# Supplementary material for: Clinical and cognitive outcomes in first-episode psychosis: focus on the interplay between cannabis use and genetic variability in endocannabinoid receptors
Source: Front Psychol. 2024 Aug 12;15:1414098. doi: 10.3389/fpsyg.2024.1414098 (PMC11348434; doi:10.3389/fpsyg.2024.1414098)
Supplement: Supplementary file 1 [file Data_Sheet_1.docx]

Supplementary Material

# Supplementary Tables

**Supplementary Table S1.** *CNR1* (rs1049353 CC vs T-car) effect on clinical and cognitive performance. Analyses were conducted using linear regressions (covaried by age, sex, and cannabis use). WAIS, Wechsler Adult Intelligence Scale; BADS, Behavioural Assessment of the Dysexecutive Syndrome; PANSS, Positive and Negative Syndrome Scale; GAF, Global Assessment of Functioning; WMS; Wechsler Memory Scale.

|  |  | *CNR1* rs1049353 |  |
| --- | --- | --- | --- |
|  | CC | T-carrier | CC vs T-car |
|  |  |  | p-value |
| Positive syndrome – PANSS | 15.13 (4.99) | 15.88 (5.59) | 0.786 |
| Negative syndrome – PANSS | 13.28 (7.62) | 17.41 (7.50) | 0.326 |
| Disorganized syndrome – PANSS | 7.00 (2.49) | 9.29 (2.08) | **0.014** |
| GAF | 50.07 (10.90) | 48.06 (11.78) | 0.947 |
| Manipulative IQ | 89.31 (16.54) | 88.17 (18.86) | 0.819 |
| Verbal IQ | 97.00 (15.11) | 90.00 (10.81) | 0.294 |
| Vocabulary test – WAIS | 10.03 (2.37) | 8.47 (1.94) | 0.123 |
| Similarities test – WAIS | 9.54 (2.77) | 8.88 (1.96) | 0.655 |
| Matrix test – WAIS | 8.31 (3.30) | 8.78 (4.40) | 0.811 |
| Block design test – WAIS | 8.89 (2.96) | 7.89 (2.85) | 0.352 |
| BADS | 16.83 (3.47) | 14.80 (4.97) | 0.083 |
| WMS | 27.91 (7.69) | 27.29 (7.51) | 0.859 |

**Supplementary Table S2.** *CNR2* (rs 2501431 AA vs G-car) on clinical and cognitive assessments. Analyses were conducted using linear regressions (covaried by age, sex, and cannabis use). WAIS, Wechsler Adult Intelligence Scale; BADS, Behavioural Assessment of the Dysexecutive Syndrome; PANSS, Positive and Negative Syndrome Scale; GAF, Global Assessment of Functioning; WMS, Wechsler Memory Scale.

|  |  | *CNR2* rs 2501431 |  |
| --- | --- | --- | --- |
|  | AA | G-carrier | AA vs G-car |
|  |  |  | p-value |
| Positive syndrome – PANSS | 15.94 (4.83) | 15.36 (5.39) | 0.782 |
| Negative syndrome – PANSS | 16.57 (7.96) | 13.65 (7.56) | 0.368 |
| Disorganized syndrome – PANSS | 8.56 (2.62) | 7.36 (2.50) | 0.223 |
| BADS | 16.21 (4.85) | 15.96 (3.85) | 0.911 |
| GAF | 47.44 (12.42) | 50.30 (10.42) | 0.613 |
| Manipulative IQ | 90.07 (17.48) | 88.21 (17.51) | 0.852 |
| Verbal IQ | 91.87 (13.32) | 95.50 (14.22) | 0.596 |
| Vocabulary test – WAIS | 9.07 (2.31) | 9.61 (2.35) | 0.714 |
| Similarities test – WAIS | 8.87 (2.23) | 9.50 (2.62) | 0.547 |
| Matrix test – WAIS | 8.72 (3.95) | 8.38 (3.70) | 0.915 |
| Block design test – WAIS | 8.53 (3.38) | 8.45 (2.72) | 0.963 |
| WMS | 26.14 (4.47) | 28.46 (8.71) | 0.371 |
